# High-Resolution Evolutionary Analysis of Within-Host Hepatitis C Virus Infection

**DOI:** 10.1093/infdis/jiy747

**Published:** 2019-01-02

**Authors:** Jayna Raghwani, Chieh-Hsi Wu, Cynthia K Y Ho, Menno De Jong, Richard Molenkamp, Janke Schinkel, Oliver G Pybus, Katrina A Lythgoe

**Affiliations:** 1 Big Data Institute, Li Ka Shing Centre for Health Information and Discovery, Nuffield Department of Medicine, University of Oxford, United Kingdom; 2 Department of Statistics, University of Oxford, United Kingdom; 3 Department of Zoology, University of Oxford, United Kingdom; 4 Department of Medical Microbiology, Amsterdam University Medical Center, the Netherlands

**Keywords:** hepatitis C virus, viral evolution, molecular epidemiology, population genetics, phylogenetics, recombination, viral replication

## Abstract

**Background:**

Despite recent breakthroughs in treatment of hepatitis C virus (HCV) infection, we have limited understanding of how virus diversity generated within individuals impacts the evolution and spread of HCV variants at the population scale. Addressing this gap is important for identifying the main sources of disease transmission and evaluating the risk of drug-resistance mutations emerging and disseminating in a population.

**Methods:**

We have undertaken a high-resolution analysis of HCV within-host evolution from 4 individuals coinfected with human immunodeficiency virus 1 (HIV-1). We used long-read, deep-sequenced data of full-length HCV envelope glycoprotein, longitudinally sampled from acute to chronic HCV infection to investigate the underlying viral population and evolutionary dynamics.

**Results:**

We found statistical support for population structure maintaining the within-host HCV genetic diversity in 3 out of 4 individuals. We also report the first population genetic estimate of the within-host recombination rate for HCV (0.28 × 10^−7^ recombination/site/year), which is considerably lower than that estimated for HIV-1 and the overall nucleotide substitution rate estimated during HCV infection.

**Conclusions:**

Our findings indicate that population structure and strong genetic linkage shapes within-host HCV evolutionary dynamics. These results will guide the future investigation of potential HCV drug resistance adaptation during infection, and at the population scale.

## Teaser

Using long-read serially sampled deep-sequenced data, we undertook a robust evolutionary analysis to formally evaluate, for the first time, whether or not HCV infection exhibits a structured viral population and the within-host HCV recombination rate.

Hepatitis C virus (HCV) is a fast-evolving RNA virus that is estimated to infect 80 million people globally [1]. Although the virus is spontaneously cleared in 15%–30% of newly infected patients, remaining individuals become chronically infected, potentially leading to severe liver disease, such as cirrhosis and liver cancer. The recent rollout of direct-acting antiviral (DAA) drugs against HCV, which can cure infection in more than 90% of cases and have considerably less toxicity than previous treatment regimens [2], has been a major breakthrough. However, treatment success varies among viral strains, host genotype, and disease status [3, 4]. In addition, DAAs remain out of reach for many individuals, especially if their infection status is unknown and/or they are living in resource-poor countries with high HCV burdens. Moreover, even though DAAs are highly effective in clearing viral infection, they do not provide long-term protection against reinfection with HCV, which may be a common occurrence in risk groups that have high rates of HCV exposure.

In order to achieve the WHO target of eliminating HCV by 2030, it will be necessary to focus on developing the most effective strategies for targeted treatment and intervention. Molecular epidemiology has become a powerful tool for understanding how viruses spread through populations, identifying predominant sources of disease transmission, and developing public health interventions [5–7]. This approach has been commonly employed for human immunodeficiency virus-1 (HIV-1) [8], but comparable studies for HCV are more challenging due to the high viral diversity observed during HCV infection, which makes it difficult to link infected individuals phylogenetically. In particular, there is a growing body of work indicating that within-host HCV diversity is maintained by multiple viral subpopulations with independent replication behavior, where distinct long-lived lineages are intermittently detected in blood plasma over time [9–13]. This observation implies that transmitted viruses are unlikely to be representative of the donor within-host HCV population sampled at a different point in time, and suggests there is potential for divergent viral strains to be transmitted from the same source individual. Consequently, to build more effective molecular epidemiology approaches for HCV, we need to understand what processes shape the within-host viral diversity, and how this impacts the evolution and spread of viral variants at the population scale.

Insights into within-host HCV evolutionary dynamics have typically been gained from short regions of the virus genome (often approximately 300 bp) and from a small number of viral sequences sampled during infection [9, 11, 14–17]. Consequently, it has been difficult to test directly and with statistical confidence if the observed HCV molecular evolutionary patterns are consistent with viral population structure (where the population comprises multiple independently replicating subpopulations with limited gene flow), in contrast to a single viral population that undergoes repeated bottlenecks (ie, fluctuating in population size over time). Furthermore, although ultradeep short-read sequence data are now commonly generated in studies of within-host viral evolution, such data are less suitable for reconstructing infection dynamics from sequences due to the large phylogenetic uncertainty associated with short reads. To address these challenges, we undertook an evolutionary analysis of full-length HCV envelope (E1E2) gene sequences serially sampled from 4 patients, which were generated previously using Pacbio SMRT sequencing [13]. The high resolution provided by long-read, high-throughput, serially sampled virus sequences is ideal for studying the within-host evolution of chronic viruses, such as HIV and HCV, which are characterized by complex and genetically diverse viral populations. Importantly, by sequencing complete gene regions at greater depth, we have sufficient power to distinguish viral variants that have emerged and persisted over the course of infection, enabling more detailed insights into the viral evolutionary dynamics.

We first investigated HCV population dynamics with a formal hypothesis-testing approach using the Bayesian statistical coalescent framework, and asked whether the observed patterns support a structured population with at least 2 independently replicating viral subpopulations, or a single population that fluctuates in size over time. For 3 individuals, we found strong statistical evidence for within-host viral population structure. Next, we estimated the within-host evolutionary rates of E1E2, which indicated considerable variation within the HCV envelope, and among individuals. Finally, we calculated genetic divergence over the course of infection, and estimated the within-host recombination rate. Notably, we found that HCV recombines considerably less frequently than HIV-1.

## METHODS

### Sequence Data

We analyzed full-length E1E2 (1680 bp) sequences from 4 individuals that were coinfected with HIV-1 and HCV. The individuals were infected with genotype 4d and were sampled longitudinally from acute to chronic infection for durations of 5 to 12 years, at average intervals of 0.66 to 1.10 years. The sequence data were generated by PacBio SMRT sequencing using stringent multiple passes to infer the circular consensus sequence reads. Further information about the sequencing and individual samples can be found in Ho et al [13].

### Evolutionary Dynamics of HCV Infection

Time-scaled phylogenies and demographic histories were estimated in BEAST v.1.8 [18] using a relaxed uncorrelated log-normal distributed molecular clock [19], a codon-structured nucleotide substitution model [20], and a Bayesian skygrid coalescent prior [21]. Bayesian phylogenetic inference methods are computationally prohibitive for large sequence datasets (>1000 sequences). Therefore we randomly subsampled 25 sequences per time point, resulting in datasets ranging from 225 to 300 sequences per individual. Two to 4 independent Markov chain Monte Carlo (MCMC) chains of 200 million steps were computed for each individual dataset to ensure that adequate mixing and stationarity had been achieved. Phylogenies were plotted with ggtree [22], and trends in within-host HCV population dynamics were inferred with ggplot2 [23] by fitting a loess regression through the N_e_τ values over time (estimated from the Bayesian skygrid model, where N_e_ is effective population size and τ is viral generation time).

The posterior trees from the above analysis were used as empirical tree distributions for estimating codon-partitioned substitution rates (CP1 + 2, first and second codon positions, and CP3, third codon position) in BEAST v1.8 [18], which were assumed as proxies for the nonsynonymous and synonymous substitution rates. The alignment was partitioned into 3 subgenomic regions: (1) E1, (2) E2 excluding the hypervariable region 1 (HVR1), and (3) HVR1. Two independent MCMC chains of 10 million steps were computed for each individual dataset.

To investigate whether the pattern of viral genetic diversity during HCV infection is better explained by a structured population characterized by 2 independently replicating populations, or by a single population with temporally varying population size, we compared 2 coalescent models: an approximation of the structured coalescent (BASTA) [24] and the Bayesian skyline coalescent [25], using the BEAST2 package [26].

To avoid biases from unequal sampling, datasets were subsampled such that each dataset contained an equal number of sequences [27]. For the structured coalescent analysis, the 2 demes were defined as follows: (1) an observed population in the blood and (2) an unobserved population, which most likely exists within the liver but could represent other compartments [10, 12]. Crucially, we assume that both subpopulations originate from the liver, because this is the main site of HCV replication, but only 1 of these subpopulations is detected in the blood at any given sampling event [10, 12]. As all HCV sequences have been sampled from the blood, there is likely insufficient information to estimate all parameters in the full structured coalescent model (eg, effective population size of the 2 demes and between subpopulation migration rates). Consequently, we constrained the between subpopulation migration rates to be equal. Biologically, this corresponds to viral lineages moving between the liver and blood at equal rates. To compare the 2 coalescent models, their corresponding marginal likelihoods were calculated using the stepping-stone sampling method [28], with an alpha parameter of 0.3 and 8 steps. The calculations were performed using the MODEL-SELECTION package of BEAST2 [26]. Because the marginal likelihood calculations require proper priors (ie, the prior distribution must integrate to 1), the Bayesian skyline coalescent prior was chosen instead of the Bayesian skygrid coalescent prior for estimating the marginal likelihood of a single population whose size varies through time. We assumed equal prior probabilities on Bayesian skyline and BASTA, as the aim was to determine which models fits the data better for each patient. Therefore, comparing marginal likelihood values is equivalent to using Bayes factors. Greater support for a structured population is indicated if a higher marginal likelihood is observed with BASTA than for the Bayesian skyline model, and vice versa.

### Divergence Over Time

For each patient, the full set of sequences (ie, not subsampled) was used to estimate the nucleotide divergence of the within-host HCV population over time using a custom Python script (https://github.com/jnarag/HCVPacbioAnalysis/blob/master/divergence.py). Specifically, divergence at a given time point was calculated as the average per-site nonsynonymous or synonymous nucleotide difference compared to the founder viral strain. If a nucleotide mutation resulted in a different amino acid, a nonsynonymous change was inferred; otherwise a synonymous change was inferred. The founder viral strain was assumed to be the consensus sequence at the first time point.

### Per-Site Amino Acid Diversity

The mean per-site amino acid diversity was estimated for each patient using a full set of sequences by implementing a custom-made python script (https://github.com/jnarag/HCVPacbioAnalysis/blob/master/AAdiversity.py). Only sites that contained no gaps were considered. Mean amino acid diversity was calculated as the number of amino acid differences among all pairs of sequences divided by the total number of unique pairs of sequences in each alignment.

### Within-Host HCV Recombination Rate

The average effective recombination rate across the 4 individuals was estimated using a custom-made Python script based on the approach outlined by Neher and Leitner [29] (https://github.com/jnarag/HCVPacbioAnalysis/blob/master/recombination.py). In brief, this method uses pairs of biallelic sites to determine how the frequency of recombinant haplotypes changes over time and distance between the sites. This approach is well suited for estimating recombination rates during within-host virus evolution, where genetic diversity is limited compared to that observed at the epidemiological scale, as it leverages the power of longitudinal sampling to identify new recombinant haplotypes.

## RESULTS

### Within-Host Population Dynamics During HCV Infection

**Figure 1.**
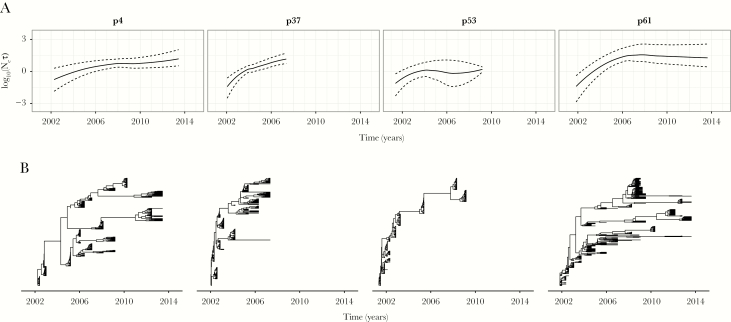
*A*, Within-host hepatitis C virus (HCV) population demographic histories and (*B*) time-scaled phylogenies for the 4 HCV/human immunodeficiency virus (HIV) coinfected individuals. *A*, Dashed lines correspond to locally estimated scatterplot *smoothing* (LOESS) regression fit through the lower and upper 95% highest posterior density estimates of N_e_τ, while the solid lines correspond to the smooth curve fitting to the mean N_e_τ over time. Abbreviations: N_e_, effective population size; τ, viral generation time, in calendar time.

Figure 1 summarizes the within-host evolutionary dynamics of the 4 individuals from acute to chronic HCV infection. Generally, the HCV population genetic diversity initially increased and subsequently stabilized in all individuals (Figure 1A). The phylogenies of 3 individuals (Figure 1B; p4, p37, and p61) were characterized by multiple viral lineages emerging in the first few years of infection, which were intermittently detected over the course of infection. This pattern was weaker in individual p37, and absent in individual p53, suggesting either not all viral lineages were sampled in the blood plasma or the degree of HCV diversification varied among individuals. Overall, these findings indicate that infection in these individuals was initiated by a single viral strain, which was followed by viral diversification and establishment, leading to the emergence and maintenance of multiple cocirculating within-host HCV lineages.

Next, we examined whether patterns of HCV genetic diversity were more consistent with a structured within-host population or with a single population whose size varied through time. Support for these alternative hypotheses was evaluated by estimating their corresponding marginal likelihoods, which can be interpreted as the probability of observing the sequence data for a given population dynamic model. For 3 individuals (p37, p53, and p61), we found stronger evidence for a structured within-host HCV population than for a single population with time-varying dynamics (Table 1). Notably, for individual p53, in spite of only 1 lineage being detected at any given sampling time point, greater support was found for a structured population (Table 1). The within-host phylogeny of p53 indicate that the internal branches have strong posterior support, in particular with the sampled populations at the last time points being relatively close in time but genetically distinct. This is likely to contribute to the observed stronger support for the structured population in this individual. This finding is in contrast to p4, where visually we observe distinct populations in the time-resolved phylogeny but do not find statistical support for a structured population; this is likely because there was little divergence in this individual at the later time points, corresponding to weak support for the different lineages. In addition, the lack of strong statistical support for a structured population in individual p4 may also reflect how the structural coalescent is defined by BASTA, which assumes a constant population size for each deme.

**Table 1.** Marginal Likelihoods Estimated Under 2 Alternative Hypotheses to Explain the Observed Hepatitis C Virus Within-Host Population Dynamics in the 4 Individuals

Individuals	BASTA	Bayesian Skyline
p4	−10279.3	−10243.3
p37	−9508.2	−9521.5
p53	−5900.1	−5949.7
p61	−10277.3	−10305.4

The 2 hypotheses are a structured population (BASTA) and a single population changing in population size over time (Bayesian skyline coalescent). Greater support for a structured population is indicated if a higher marginal likelihood is observed with BASTA than for the Bayesian skyline coalescent model, and vice versa.

### Molecular Evolution of the HCV Envelope Region

The codon partitioned substitution rates (CP1 + 2, first and second; CP3, third) for E1, E2 excluding HVR1, and HVR1 are summarized in Table 2. The mean CP1 + 2 rates varied considerably both among gene regions and individuals, with the HVR1 exhibiting the highest rate of evolution, ranging from 1.5 to 4.8 × 10−^2^ substitutions/site/year. In individual p4, an elevated CP1 + 2 evolutionary rate was observed in the E1 region, corresponding to an accumulation of substitutions that was 7–10 times faster relative to the other individuals (Table 2). A similar difference in CP1 + 2 evolutionary rates was also observed in E2 with individuals p4 and p37 accumulating substitutions 5–11 times faster than p53 and p61. As noted previously, the fastest evolving region within E1E2 was the HVR1 [9, 15, 30, 31], which is consistent with stronger diversifying selection acting upon this region, most likely reflecting its role in evading humoral immunity.

**Table 2.** Mean Within-Host Evolutionary Rates in Hepatitis C Virus Envelope Glycoprotein

		Within-Host Evolutionary Rate, 10^–2^ substitutions/site/year (95% Credible Interval)			
		Individuals			
Gene Region	Partition	p4	p37	p53	p61
E1	CP1 + 2	1.942 (1.467, 2.465)	0.193 (0.127, 0.257)	0.240 (0.132, 0.347)	0.211 (0.146, 0.278)
E2	CP1 + 2	1.791 (1.303, 2.248)	2.233 (1.602, 2.834)	0.363 (0.237, 0.504)	0.209 (0.159, 0.265)
HVR1	CP1 + 2	3.361 (1.805, 6.255)	4.841 (2.855, 6.727)	3.540 (1.113, 8.772)	1.475 (1.115, 1.921)
E1	CP3	0.476 (0.224, 0.747)	0.187 (0.119, 0.255)	0.202 (0.109, 0.295)	0.168 (0.129, 0.210)
E2	CP3	0.572 (0.425, 0.722)	0.443 (0.317, 0.584)	0.221 (0.135, 0.314)	0.257 (0.210, 0.313)
HVR1	CP3	0.726 (0.272, 1.123)	2.584 (0.927, 8.089)	0.896 (0.191, 1.865)	0.592 (0.271, 0.964)
Envelope	Total	2.390 (2.014, 2.824)	2.514 (2.030, 3.027)	0.749 (0.503, 1.037)	0.746 (0.650, 0.841)

Differences in CP3 rates were less marked, with the exception of a higher substitution rate in HVR1 observed for individual p37 (although this is associated with large statistical uncertainty; Table 2). The overall substitution rates of E1E2 suggest that the within-host HCV populations in the 4 individuals have evolved at varying rates, with slower rates observed in individuals p53 and p61 than in individuals p4 and p37 (Table 2). These observations have most likely arisen from individual variation in host immune responses and the underlying ecology of the within-host viral population.

**Figure 2.**
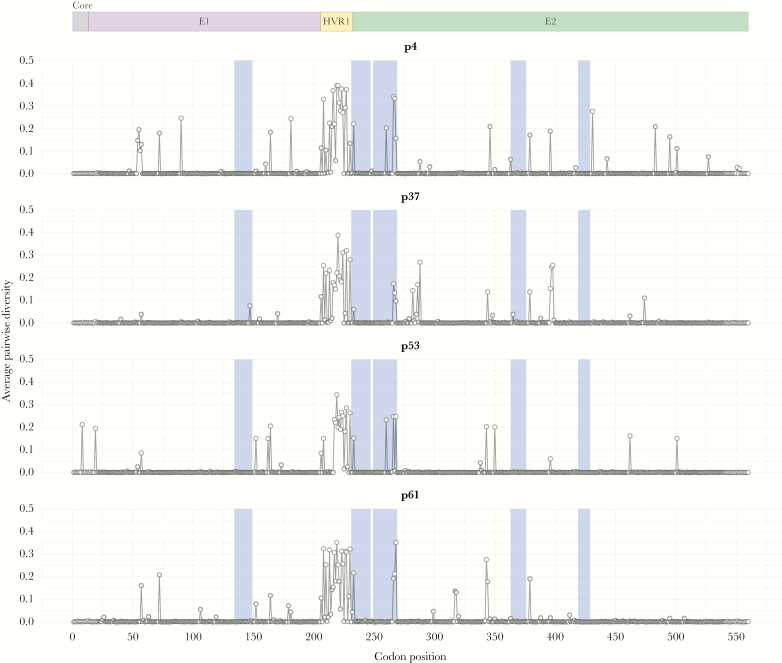
Mean amino acid diversity per site. The genomic regions (Core, E1, HVR1, and E2) are indicated above the plot. The shaded regions indicate putative neutralizing antibody epitopes. This information was collated from the Immune Epitope Database, which corresponds to regions in hepatitis C virus (irrespective of genotype or subtype) that have been experimentally confirmed to elicit an antibody response.

Figure 2 illustrates the per-site amino acid diversity in E1E2 in the 4 individuals. Most of the E1E2 was conserved in all 4 individuals. As expected, the HVR1 was characterized by the greatest amino acid diversity. There is some evidence of elevated diversity at the neutralizing antibody epitope located in E2 at positions 249–269 (H77 reference 428–447), where the per-site diversity was approximately 0.25 or greater in 3 out of 4 individuals. However, without information on the specific immune response elicited in these individuals, it is difficult to determine if this pattern was driven by viral immune evasion. In individual p37, 2 regions in E2 exceeded a per-site diversity of 0.25 (Figure 2; sites 288 and 398), which might explain the comparatively higher CP1 + 2 rate observed in the E2 region of this individual (Figure 2 and Table 2). Interestingly, position 398 falls within an N-linked glycosylation site, which exhibits considerable variation during infection, possibly reflecting ongoing viral evasion from host immune responses.

### Population Genetic Analysis of HCV Infection

**Figure 3.**
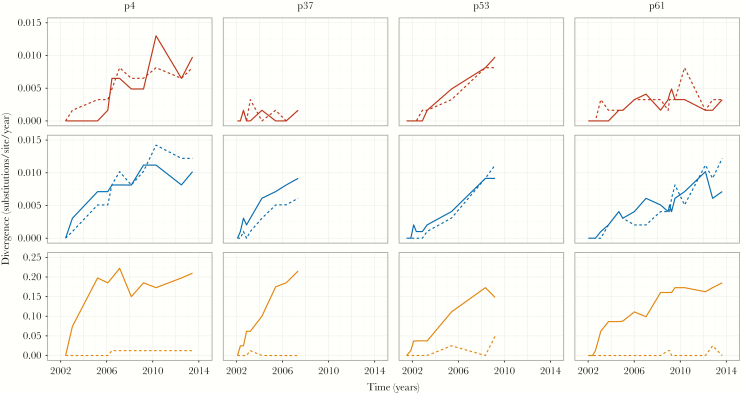
Divergence over time for each gene region (E1, E2, and HVR1 on top, middle, and bottom rows, respectively). Solid lines correspond to nonsynonymous divergence, while dashed lines correspond to synonymous divergence. Note, scales of the y-axes for HVR1 are different from those of E1 and E2.

We also analyzed the full sequence dataset obtained for each individual (ranging from 5479 to 9122 sequences across all time points per individual) using simpler, population genetic summary statistics. First, we measured the divergence of the within-host viral population in 3 different gene regions for all individuals (Figure 3). The overall patterns of divergence are consistent with the evolutionary rates in Table 2. In general, E1 and E2 accumulated nonsynonymous and synonymous substitutions at similar rates within individuals, while the nonsynonymous divergence in HVR1 was an order of magnitude higher. This strongly suggests that HVR1 is mainly under immune-mediated selection, while the rest of E1E2 experiences purifying selection (Figure 3). The apparent lack of synonymous divergence in HVR1 is largely due to the differences in the scales of the y-axes for each subgenomic region. On closer inspection, the synonymous divergence in HVR1 was comparable to E1 and E2, although the pattern through time was more stochastic (Supplementary Figure 1). Lastly, while the gene regions largely diverged at an approximately constant rate over time, in individuals p4 and p61 we found that the nonsynonymous divergence in HVR1 slowed down after 1 to 2 years of infection (Figure 3).

The average within-host recombination rate during HCV infection, across all individuals, was estimated to be 0.28 × 10^−7^ recombinations/site/day (interquartile range: 0.13–1.05 × 10^−7^). This is significantly lower than the inferred within-host substitution rate, which ranged from 2.05 to 8.21 × 10^−5^ substitutions/site/day. Furthermore, the HCV recombination rate is approximately 2 orders of magnitude lower than that observed for HIV-1 (estimated using the same method) [29]. The low within-host recombination rate indicates that strong linkage effects will influence the viral evolutionary dynamics during infection. Our estimate of recombination rate could be inflated by PCR recombination. If so, the true rate of within-host recombination will be even lower than that estimated here, further supporting our conclusions that strong linkage effects are likely to dominate HCV within-host evolution.

## DISCUSSION

By examining the evolution of full-length E1E2 sequences from acute to chronic infection, we found that chronic HCV infection was consistent with independently replicating viral subpopulations [10, 12, 32] that were established from a single infecting viral strain, but not all were necessarily detected in the blood at all time points. We also found marked variation in the rates of evolution across the different regions of the envelope, and among individuals, for both synonymous and nonsynonymous mutations, combined with a very low rate of within-host recombination.

Given that longitudinal sampling of both the liver and blood from untreated HCV-infected individuals is unlikely to be feasible, or ethical, it has been difficult to test directly if chronic HCV infection is maintained by a structured population. To address this using an evolutionary approach, we assessed formally whether the observed within-host HCV population dynamics in the 4 individuals support a structured viral population characterized by 2 demes, where only of 1 these subpopulations is observed in the blood at any point in time, or with a single population whose population size varies over time. This analysis was possible due to the comparatively long reads in this dataset, which provided sufficient statistical power to evaluate the 2 alternative hypotheses of HCV population dynamics. For 3 individuals, we found a statistically better fit for a structured population. Although this result overall supports our original hypothesis, it is unclear why we did not find evidence for a structured population in 1 of the individuals (p4) despite observing cocirculation of multiple viral lineages during chronic infection. The lack of divergence at later time points in this individual might explain this apparent discrepancy. In addition, more complex within-host HCV population dynamics than assumed by the structured coalescent, such as one or more viral subpopulations undergoing changes in population size, could explain the lack of support for a structured viral population. Notably, our finding of significant statistical support for a structured viral population for individual p53, where the within-host phylogeny was characterized by a single viral lineage at any point in time, underlines the importance of formal statistical testing to evaluate alternative hypotheses about the viral population dynamics, and highlights the potential problems of making inferences from visual inspection of within-host phylogenies alone.

Molecular evolutionary analyses of the E1E2 revealed very high rates of nonsynonymous divergence at the HVR1 region, which is consistent with this small genomic region undergoing strong immune-mediated selection [15, 30, 31, 33, 34]. In contrast, the rest of E1E2 is largely characterized by purifying selection. Although similar conclusions have been reported previously [9, 15, 30, 31], the combination of frequent sampling during HCV infection and long-read sequence data has enabled us to robustly compare the rates of molecular evolution among the different gene regions in the HCV envelope both within and between individuals.

In this study we also report, for the first time, an estimate of the within-host recombination rate of HCV during infection that can be directly compared with other evolutionary estimates. In contrast to HIV-1 [29], recombination appears to be a weak evolutionary force during within-host HCV evolution. This helps to explain why considerably fewer circulating recombinant forms are observed at the epidemiological scale for HCV than for HIV-1, even though HCV is more transmissible and mixed infections with distinct genotypes are relatively common. Furthermore, the within-host recombination rate is substantially lower than the overall substitution rate of E1E2, indicating that strong linkage effects are likely to shape the within-host viral evolutionary dynamics; selection is expected to be less effective in nonrecombining populations due to clonal interference [35] and background selection [36–38], thus reducing the rate of fixation of beneficial mutations in the population. A structured population can also limit viral adaptation if migration rates between viral subpopulations are low [39], since beneficial mutations will be restricted to the subpopulations in which they emerged, thereby preventing them from sweeping through the global population. Consequently, these evolutionary dynamics suggests that although drug resistance mutations might emerge during infection, fixation within individuals and transmission between individuals could be restricted.

To fully understand the extent to which within-host HCV populations are structured, and the effect that this, combined with low rates of recombination, has on HCV during infection, will require additional viral sequence data that has been serially sampled from a greater number of individuals. As well as collecting key clinical information, such as HLA background, viral load, and antibody responses, greater priority should be given to long-read, deep-sequenced data that spans the whole virus genome, because this will give the necessary power to determine whether, and how many, distinct viral subpopulations exist. This will be especially important for determining whether viral population structure is associated with strong selection or genetic drift, and will help to elucidate the relative contribution of cell-mediated and humoral immunity during HCV infection.

## Supplementary Data

Supplementary materials are available at *The Journal of Infectious Diseases* online. Consisting of data provided by the authors to benefit the reader, the posted materials are not copyedited and are the sole responsibility of the authors, so questions or comments should be addressed to the corresponding author.

## Supplementary Material

Supplementary Figure S1

## Notes

***Financial support.*** This work was supported by the Wellcome Trust and the Royal Society (grant number 107652/Z/15/Z); and the European Research Council (ERC) under the European Union’s Seventh Framework Programme (grant number FP7/2007-2013/ERC grant agreement no. 614725-PATHPHYLODYN).

***Potential conflicts of interest.*** All authors: No reported conflicts of interest. All authors have submitted the ICMJE Form for Disclosure of Potential Conflicts of Interest. Conflicts that the editors consider relevant to the content of the manuscript have been disclosed.
