# Supplementary material for: High-Resolution Evolutionary Analysis of Within-Host Hepatitis C Virus Infection
Source: J Infect Dis. 2019 Jan 2;219(11):1722–9. doi: 10.1093/infdis/jiy747 (PMC6500553; doi:10.1093/infdis/jiy747)
Supplement: Supplementary Figure S1 [file jiy747_suppl_supplementary_figure_s1.docx]

Figure S1: Summary of HVR1 divergence – Overall, nonsynonymous, and synonymous divergence shown from left to right for each of the four individuals.
